# Suppressed Programmed Death 1 Expression on CD4^+^ and CD8^+^ T Cells in Psoriatic Patients

**DOI:** 10.1155/2017/5385102

**Published:** 2017-10-17

**Authors:** Joanna Bartosińska, Ewelina Zakrzewska, Dorota Raczkiewicz, Joanna Purkot, Anna Michalak-Stoma, Małgorzata Kowal, Dorota Krasowska, Grażyna Chodorowska, Krzysztof Giannopoulos

**Affiliations:** ^1^Department of Dermatology, Venereology and Paediatric Dermatology, Medical University of Lublin, Lublin, Poland; ^2^Experimental Hematooncology Department, Medical University of Lublin, Lublin, Poland; ^3^Institute of Statistics and Demography, Warsaw School of Economics, Warsaw, Poland

## Abstract

Psoriasis is a chronic inflammatory disease mediated by T cell immunity. Programmed death 1 (PD-1), a coinhibitory receptor, plays an important role in immune regulation and maintaining peripheral tolerance. The aim of the study was to compare the expression of PD-1 on the peripheral T cells between psoriatic patients and healthy controls. The study included 75 psoriatic patients and 52 healthy volunteers. The percentages and absolute numbers of CD3^+^, CD4^+^, CD8^+^, CD4^+^PD-1^+^, and CD8^+^PD-1^+^ T cells were analyzed using flow cytometry. The absolute numbers and percentages of CD4^+^PD-1^+^ and CD8^+^PD-1^+^ T cells were significantly decreased in the psoriatic patients in comparison with the control group. No significant correlations were found between the absolute numbers and percentages of CD4^+^PD-1^+^ or CD8^+^PD-1^+^ T cells and clinical characteristics of psoriasis. Decreased PD-1 expression on the T cells may be responsible for impaired negative regulation of immune response in psoriasis pathogenesis.

## 1. Introduction

Psoriasis is a chronic inflammatory disease with the substantial involvement of T lymphocytes, where already activated T cells in the blood and skin are bound to cause cutaneous inflammation and keratinocyte hyperproliferation [1]. This pathological process has been explained before, but the role of certain regulatory mechanisms responsible for the immune tolerance in this disease needs to be further researched.

Various mechanisms, either contact dependent or related to secretion of some soluble factors, are employed to suppress immune responses. A number of surface molecules on T cells which regulate their state of activation have been identified.

The activation of T cells requires at least two signals: the first signal is an interaction between the T cell receptor (TCR) and major histocompatibility complex (MHC) on the antigen-presenting cell (APC), and the second signal is costimulation which could be stimulatory or inhibitory. One of the most important coinhibitory signals is an interaction between the programmed death 1 (PD-1), a molecule belonging to the CD28 family which is expressed on the T cells, B cells, macrophages, and PD-L1 (PD-ligand 1) or PD-L2, on the APC. Binding of ligands to PD-1 leads to downregulation of T cell activity [2–4].

PD-1, a checkpoint inhibitor, is vital for the immune regulation and tolerance [4]. Its blockage is known to provoke a shift of the cellular reactivity towards the proinflammatory response. The role of PD-1 expression in psoriasis pathogenesis has not been entirely explained so far. Therefore, we have made an attempt to determine the expression of PD-1 on CD4^+^ and CD8^+^ T cells from the peripheral blood of psoriatic patients. It is likely that the absence of negative costimulation from PD-1 is responsible for continuous T cell activation and sustained skin inflammation in psoriasis, which may also contribute to the systemic nature of the disease.

## 2. Materials and Methods

### 2.1. The Study Group

The study group consisted of 75 psoriatic patients hospitalized in the Department of Dermatology, Venereology and Pediatric Dermatology Medical University of Lublin, Poland. The inclusion criteria were as follows: the duration of psoriasis for at least one year, active psoriatic skin lesions, and age at least 18 years. The exclusion criteria were as follows: cardiovascular, cerebrovascular, hematologic, hepatic or renal disease, neoplasm, chronic viral infections, erythrodermic, pustular or guttate psoriasis, addiction to drugs, and systemic antipsoriatic treatment.

The control group included 52 healthy volunteers, age- and gender-matched to the psoriatic group.

Informed consent was obtained from all the participants, and the study was approved by the Local Ethics Committee at the Medical University of Lublin (KE-0254/81/2015).

### 2.2. Assessment of Psoriasis Severity

The severity of psoriasis was assessed with PASI (Psoriasis Area and Severity Index), BSA (Body Surface Area), IGA (Investigator Global Assessment), and DLQI (Dermatology Life Quality Index). Psoriatic fingernail plate changes were assessed using NAPSI 80 (Nail Psoriasis Severity Index 80). We also analyzed the duration and the age of psoriasis onset.

### 2.3. Flow Cytometry Analysis of Peripheral Blood Mononuclear Cell Populations and Expression of PD-1

Flow cytometry analysis was performed in the Experimental Hematooncology Department at the Medical University of Lublin, Poland, with professionally trained and experienced staff in such analyses in patients with lymphoproliferative diseases.

Eight mL of peripheral venous blood from the psoriatic patients and healthy volunteers were collected into anticoagulated tubes. We isolated mononuclear cells using density gradient centrifugation on Ficoll-Hypaque (Biochrom AG, Berlin, Germany). Interphase cells were removed, washed twice in phosphate-buffered saline (PBS) without Ca^2+^ and Mg^2+^ and resuspended in RPMI 1640 containing 2% human albumin. The viability of obtained PBMCs was always >95%, as determined by trypan blue staining. Viable cells were quantified in a Neubauer chamber. 5 × 10^5^ cells were incubated for 20 min. at room temperature with fluorochrome-labeled monoclonal antibodies (Mabs): anti-CD3-PerCP (Becton Dickinson), anti-CD4-FITC (Becton Dickinson), anti-CD8-PE (Becton Dickinson), and anti-PD-1-APC (clone MIH4) (Becton Dickinson). Approximately 100,000 stained cells in each sample were analyzed by flow cytometry using a FACS Canto II flow cytometer (BD Biosciences, San Jose, CA, USA). Unstained cells were used as a negative control (for each patient). Data analysis was accomplished by using FACS Diva 8.0.

**Figure 1:**
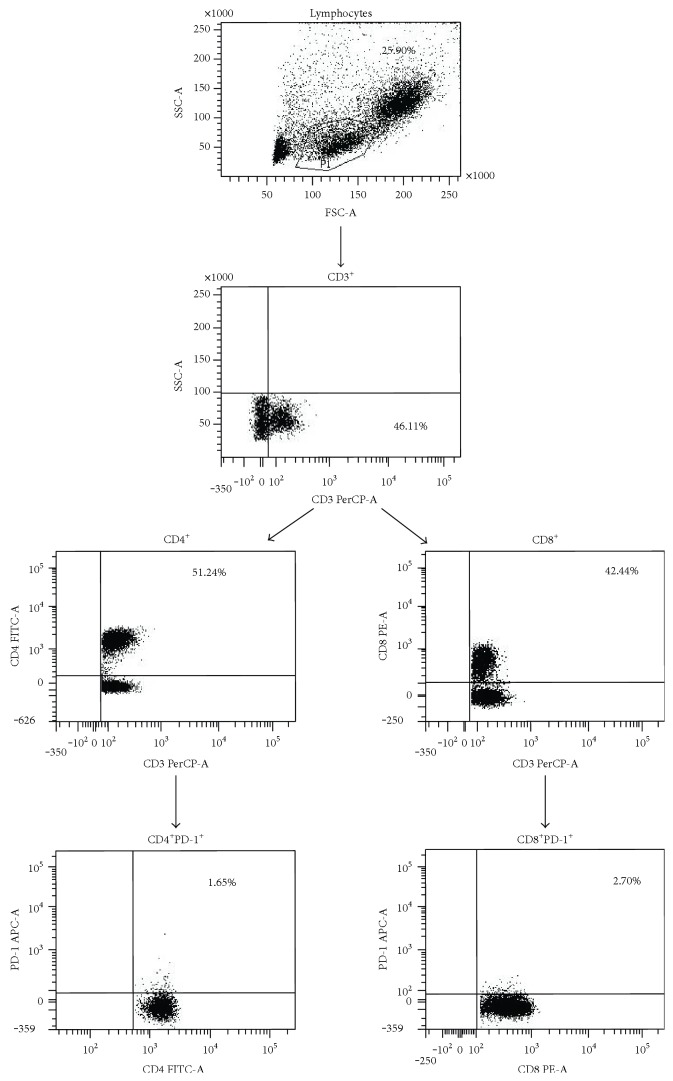
Full gating strategy for flow cytometry analysis of PD-1 expression on T cells from a psoriatic patient.

**Figure 2:**
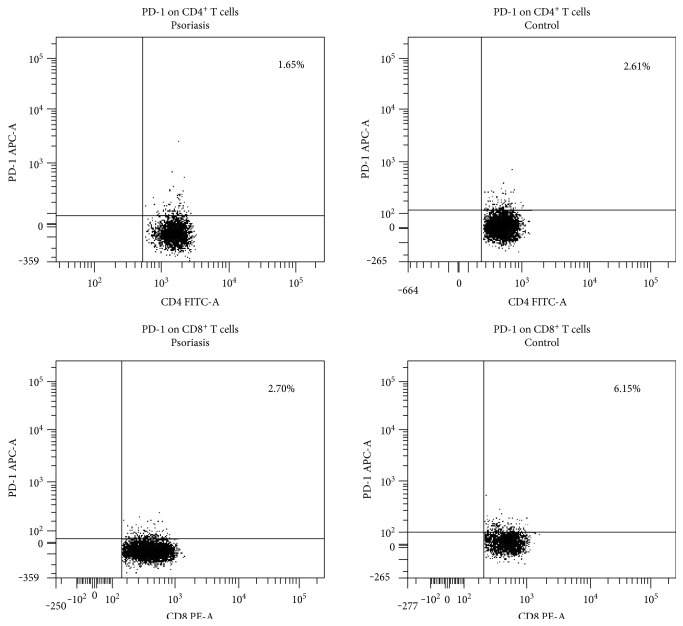
Representative flow cytometry analysis of PD-1 expression on CD4^+^ and CD8^+^ T cells from psoriatic patients and healthy controls.

For each person, lymphocytes were identified and gated from PBMCs by setting appropriate forward and side scatter parameters. Next, 3 steps of gating were applied in flow cytometry analysis (Figure 1() shows it for one exemplary patient with psoriasis). The first dot plot showed CD3^+^ cells selected from all lymphocytes, and as a result, we obtained percentages of CD3^+^ cells of all lymphocytes. The two gates on the second step of the cytometry were selected separately: one gate CD4^+^ T cells and the other gate CD8^+^ T cells, resulting in percentages of CD4^+^ and CD8^+^ T cells, respectively, for all CD3^+^ cells. The two gates on the third step of the cytometry were selected separately: one gate CD4^+^PD-1^+^ T cells and the other gate CD8^+^PD-1^+^ T cells, resulting in percentages of CD4^+^PD-1^+^ T cells and CD8^+^PD-1^+^ T cells for CD4^+^ and CD8^+^ T cells, respectively (Figure 2() shows 2 exemplary persons: one patient with psoriasis and one healthy volunteer).

Then, for each person, we calculated the absolute number of CD3^+^ cells, multiplying the total number of lymphocytes by percentages of CD3^+^ (the result from the first step of gating). Then, we calculated the absolute numbers of CD4^+^ and CD8^+^ T cells, multiplying the absolute number of CD3^+^ cells (calculated above) by percentages of CD4^+^ and CD8^+^ T cells, respectively (the results from the second step of gating). At the end, we calculated the absolute numbers of CD4^+^PD-1^+^ and CD8^+^PD-1^+^ T cells, multiplying the absolute numbers of CD4^+^ and CD8^+^ T cells, respectively (calculated above) by percentages of CD4^+^PD-1^+^ and CD8^+^PD-1^+^ T cells, respectively (the results from the third step of gating).

### 2.4. Statistical Analysis

Data of both absolute numbers (cells/*μ*L) and percentages of PBMCs and PD-1 expression were statistically analyzed in the Institute of Statistics and Demography, Warsaw School of Economics, Poland, using SPSS and STATISTICA softwares.

We used a *t-*test to compare age and stochastic independence and *χ*^2^ test to compare gender between the psoriatic patients and the control group.

We analyzed the clinical data of the psoriatic patients. Mean values (*M*) and standard deviations (SD) were estimated for continuous variables or absolute numbers (*n*) and relative numbers (%) of occurrence of items for categorical variables.

Comparisons of number and percentages of T cells and expression of PD-1 between the psoriatic patients and the control group were performed using logistic regression models. We estimated odds ratios of psoriasis occurrence versus control group (psoriasis—yes versus psoriasis—no), with independent variables: the absolute numbers (the first models) and percentages of PBMCs and PD-1 expression (the second models). In order to interpret the logistic regression analyses' results, we calculated OR-1 and expressed them in %. As independent variables are continuous, we obtained an average percentage change in risk of psoriasis (in plus or in minus) if independent variable increased about 1 unit (1 cell/*μ*L in the first model or 1 percentage in the second model).

We used the Pearson correlation coefficient (*r*) to investigate mutual correlations of the number and percentages of PD-1 expression between CD4^+^ and CD8^+^ T cells, as well as correlations of the number and percentages of T cells and PD-1 expression with clinical data in psoriatic patients.

On the basis of the central limit theorem, it is commonly assumed in practice that parameters' estimators of sample size over 60 (even 30) sample units are asymptotically normal distributed.

In statistical tests, we agreed to a probability up to 0.05 in making an error in rejection of true null hypothesis, which assumes no dependence between variables.

## 3. Results

### 3.1. Characteristics of the Study Group

The psoriatic patients and the control group did not significantly differ in age (*t* = 0.233, *p* = 0.785) and sex (*χ*^2^ = 0.381, *p* = 0.537). Age and sex of the psoriatic patients compared to the control group, as well as patients' clinical data for psoriasis duration and severity, were presented in Table 1.

### 3.2. Comparison of the PBMC Population Distribution and PD-1 Expression between the Psoriatic Patients and Healthy Controls

Table 2 presents the distribution of PMBC populations and PD-1 expression in the psoriatic patients compared to the control group and the logistic regression analysis results of the psoriasis' odds ratios compared to the healthy controls.

The absolute numbers and percentages of the CD3^+^ and CD8^+^ cells were significantly increased in the psoriatic patients in comparison with the control group. The estimated odds of psoriasis increased by 0.1% if the number of CD3^+^ increased by 1 cell/*μ*L and by 0.4% if the number of CD8^+^ increased by 1 cell/*μ*L, on average. The estimated odds of psoriasis increased by 5.1% if percentages of CD3^+^ cells increased by 1% and by 3.9% if percentages of CD8^+^ increased by 1%, on average.

The absolute numbers and percentages of the CD4^+^ T cells did not significantly differ between the psoriatic patients and the healthy controls.

The absolute numbers and percentages of the CD4^+^PD-1^+^ and CD8^+^PD-1^+^ T cells were significantly decreased in the psoriatic patients in comparison with the control group. The estimated odds of psoriasis decreased by 3.3% if the number of CD8^+^PD-1^+^ increased by 1 cell/*μ*L, by 36.9% if percentages of CD4^+^PD-1^+^ cells increased by 1%, and by 29.5% if percentages of CD8^+^PD-1^+^ cells increased by 1%, on average.

### 3.3. Analysis of Mutual Correlations between PD-1 Expression on CD4^+^ and CD8^+^ T Cells in the Psoriatic Patients

We observed a positive correlation between the absolute numbers of CD4^+^PD-1^+^ and CD8^+^PD-1^+^ T cells (*r* = 0.534, *p* < 0.001), as well as between the percentages of CD4^+^PD-1^+^ and CD8^+^PD-1^+^ T cells (*r* = 0.347, *p* = 0.002).

### 3.4. Analysis of Correlations between the PBMC Population Distributions and PD-1 Expression and Clinical Data in the Psoriatic Patients

We correlated the PBMC population distributions and PD-1 expression with characteristic of psoriasis, that is, duration and the age of the disease onset and psoriasis severity expressed by PASI, BSA, IGA, NAPSI, and DLQI (Table 3).

The absolute cell number of CD3^+^ correlated negatively with PASI and NAPSI 80.

The absolute number and percentages of CD4^+^ T cells correlated negatively with PASI and IGA, and the absolute cell number of them also correlated negatively with BSA and NAPSI 80.

The absolute number of CD8^+^ T cells did not correlate with any clinical data, but the percentages of them correlated positively with PASI, BSA, and IGA.

The absolute number of CD4^+^PD-1^+^ T cells correlated negatively only with PASI, and the absolute number of CD8^+^PD-1^+^ T cells correlated negatively only with the age of psoriasis onset.

The percentages of CD4^+^PD-1^+^ and CD8^+^PD-1^+^ T cells did not correlate with any clinical data of the psoriatic patients.

## 4. Discussion

The issue of the expression of negative costimulatory molecule PD-1 on the peripheral CD4^+^ and CD8^+^ T cells was interesting enough for us to launch an innovative research into its role in the psoriasis pathogenesis. The results of our investigations showed significantly decreased protein PD-1 expression on both CD4^+^ and CD8^+^ T cells and a positive correlation between the CD4^+^PD-1^+^ and CD8^+^PD-1^+^ T cells. It is well known that the peripheral blood T cells, including CD4^+^ and CD8^+^ T cells, are involved in psoriasis following a persistent stimulation by immunogens [5]. It has been found that in the psoriatic skin lesion the CD4^+^ T cells accumulate mainly in the dermis, whereas the CD8^+^ T cells are found in the epidermis [6]. Sigmundsdóttir et al. [7] observed a significantly higher frequency of CD8^+^ T cells in the blood and their positive correlation with PASI.

According to our study, the frequency of CD3^+^ and CD8^+^, but not CD4^+^ T cells, was significantly higher in the psoriatic patients than in the healthy controls. Similar to the results published by Sigmundsdóttir et al. [7], we have found a positive correlation between the percentages of CD8^+^ T cells and PASI, but also by IGA. The increased numbers of circulating inflammatory CD8^+^ T cells may confirm their active role in the psoriasis pathogenesis. Nevertheless, in our study, the absolute number of CD8^+^ T cells negatively correlated with the patient's age at the age of psoriasis onset. CD4^+^ T cells are believed to be necessary for the initiation of psoriatic skin lesions [7]; however, the exacerbation of psoriasis is observed in acquired immunodeficiency syndrome (AIDS) upon CD4^+^ T cell depletion [8]. In our study, the negative correlations between the percentages and absolute number of CD4^+^ T cells and severity of psoriasis measured by PASI and IGA were observed.

Although many studies on psoriasis pathogenesis have been conducted so far, the role of the PD-1/PD-L1 pathway in the disease has not been explained yet.

Considering the fact that PD-1 plays a role in a normal immune response silencing, its reduced expression may contribute to the chronicity and frequent recurrence of psoriasis. Therefore, the reduced expression of PD-1 on T cells could be a marker of the disease activity and failure of the feedback mechanism which would be able to prevent the immune overstimulation and autoimmunity.

The results of our study might suggest deregulation of immune suppression mechanisms, which may lead to abnormal persistent T cell activation in psoriasis. Ferenczi et al. [9] found that in psoriatic patients, most of the lesional T cells expressed the three primary activation markers (CD25, CD69, and HLA-DR), whereas psoriatic blood T cells were characterized by high CD25 expression. Lymphocyte activation through analysis of CD25 and CD69 expression in psoriatic patients was also determined by Porto Ferreira et al. [10]. Higher percentages of activated (CD25^+^ and CD69^+^) cells were detected in both CD4^+^ and CD8^+^ lymphocyte subpopulations in the blood of psoriatic patients. Although the results compared to controls were significant only for the percentage of CD25^+^ cells in the CD8^+^ T cell subpopulation, there was a trend to increased expression of CD25 and CD69 in both CD4^+^ and CD8^+^ T cells. The presence of activation cells has been observed in the early stages of the disease, and even before clinically apparent lesions, activation molecules are probably engaged in lymphocyte migration and recruitment [11, 12]. These results indicate that T cells which express activation markers are involved in the initiation and progression of psoriasis lesions [10]. Therefore, deregulated PD-1 signaling pathway might result in sustaining chronic inflammation and promotion of autoinflammatory changes.

In their quantitative real-time RT-PCR, Western blotting, and immunohistochemistry studies, Kim et al. [13] found decreased expression of PD-L1 as well as PD-L2 in psoriatic epidermis compared to the healthy controls. Interestingly, the authors compared the results not only with the normal skin but also with the skin samples collected from the patients with allergic contact dermatitis, pityriasis rosea, and lichen planus in which no decreased expressions of PD-L1 and PD-L2 were present. The authors suggested that decreased expression of PD-L1 and PD-L2 could result from impairment of the Treg function in psoriasis and it could allow continuous T cell activation. Expression of PD-L1 and PD-L2 on the endothelial cells reduces the influx of T cells into the inflamed tissues. Thus, PD-1 reduced expression on CD4^+^ and CD8^+^ T cells may contribute to the increased flow to the tissues and to the synthesis of cytokines. It is probable that in psoriasis PD-L1 and PD-L2 on keratinocytes may interact with PD-1 on T cells and modulate the immune response. Kim et al. [13] suggested that PD-L1 and PD-L2 could possibly be used in psoriasis treatment, that is, as a topical drug which would be able to normalize their epidermal expression.

In the group of 20 psoriatic arthritis patients, Peled et al. [14] found that the percentages of CD3^+^PD-1^+^ T cells were higher in the patients than in the healthy controls. Similar to the results of our study, the authors did not find any correlation between the level of PD-1 expressing T cells and PASI. Although in our study the absolute number and percentages of CD4^+^ T cells correlated negatively with PASI and IGA as well as the percentages of CD8^+^ T cells correlated positively with PASI, no correlation was observed between CD4^+^PD-1^+^ or CD8^+^PD-1^+^ T cells and the clinical characteristics of psoriasis. Therefore, it might be speculated that a decreased expression of PD-1, regardless of its level, is a triggering factor of Th1 and Th17 activation.

Recently Shin et al. [15] observed decreased levels of PD-1^+^ blood follicular helper T cells (T_FH_) in the group of 28 psoriatic patients. The absolute number of CXCR5^+^PD-1^+^ T_FH_ cells correlated positively with the disease duration. The authors also did not find any correlation with PASI. Wang et al. [16] in the group of psoriatic patients found increased frequency and activation of T_FH_ (confirmed by the higher expression of their two important surface markers ICOS (inducible T cell costimulatory) and PD-1). Niu et al. [17] observed also higher levels of circulating CD3^+^CD4^+^CXCR5^+^ cells; CD3^+^CD4^+^CXCR5^+^ICOS^+^, CD3^+^CD4^+^CXCR5^+^PD-1^+^, and CD3^+^CD4^+^CXCR5^+^ICOS^+^PD-1^+^T_FH_ cells; and CD19^+^IgD^+^CD27^−^naive B and CD19^+^CD86^+^-activated B, but lower levels of CD19^+^IgD^+^CD27^+^ preswitch and CD19^+^IgD^−^CD27^+^ postswitch memory B cells compared with healthy donors. Importantly, the observed frequencies of these T cell subpopulations were very low.

PD-1 expression is known as a possible diagnostic marker in malignancy, including cutaneous carcinogenesis. Upregulated PD-1 and PD-L1 were observed in several human cancer types, including melanoma and hematological neoplasms [2, 3]. Their higher expression in skin lymphomas may be of special interest. Interestingly, the assessment of PD-1 expression may appear to be useful in differential diagnosis between the Sezary syndrome and various erythrodermic inflammatory dermatoses, including psoriasis. Çetinözman et al. [18] observed that in psoriatic erythroderma patients, the median percentages of the PD-1^+^ T cells was 20% in comparison to 90% in the SS patients.

Moreover, the agents with anti-PD-1 action may inhibit Treg or promote the shift of Treg into Th17. PD-1 inhibitors are used in various human cancers and malignant neoplasm treatment in order to block the interaction between PD-1 and PD-L1 to increase antitumor immunity [4]. In the light of Dulos et al.'s [4] study, it is probable that augmentation of Th1 and Th17 responses during anti-PD-1 treatment is responsible for resistance to PD-1 blockade. Since IL-17 might be involved in tumor growth as a proangiogenic factor, some authors suggest a possible synergistic effect of the anti-PD-L1/Th17 axis for cancer treatment.

Interestingly, it has been observed that patients treated with nivolumab, a human anti-PD-1 antibody, may develop skin rashes, dermatitis, and psoriasiform dermatitis. Moreover, PD-1 genetic deficiency in mice (*pdcd1^−/−^*) results in spontaneous development of arthritis, dilated cardiomyopathy, or lupus-like autoimmune disease [19, 20]. Imai et al. [20] found that in PD-1-deficient (PD-1KO) mice after imiquimod application both IL-17A and IL-22 were enhanced and resulted in increased dermal inflammation, epidermal acanthosis, and neutrophilic abscess formation. Their study also showed that PD-1 blockade by specific antibody markedly exacerbated psoriasiform dermatitis in mice.

Moreover, some recent reports present the development of severe psoriasis in patients treated with immunotherapy using PD-1 inhibitors. Chia and John [21] presented a 74-year-old man with metastatic lung cancer in whom severe psoriasis flare developed after two cycles of a PD-1 inhibitor (pembrolizumab) treatment. Psoriasis was resolved after the cessation of immunotherapy, topical corticosteroid, and phototherapy application. Similarly, Matsumura et al. [22] observed an exacerbation of psoriasis in an 87-year-old patient after two cycles of nivolumab for metastatic melanoma.

Therefore, in our study, we have also performed logistic regression analysis which has shown that estimated odds of psoriasis decreased by 36.9% if CD4^+^PD-1^+^ T cells increased by 1% and by 29.5% if CD8^+^PD-1^+^ T cells increased by 1%, compared to the control group. The finding confirms the role of negative costimulation in preventing psoriasis development and shows that patients treated with PD-1 inhibitors will require dermatological consultations and treatment.

Since disturbed regulatory mechanisms of the immune response and immune tolerance are observed in psoriasis, the assessment of the PD-1/PD-L1 pathway in the pathogenesis of psoriasis needs in-depth investigation. Pinpointing the role of PD-1 expression in psoriasis could provide unambiguous answers to whether PD-1 triggers the onset of psoriasis, favouring its development or both.

## Figures and Tables

**Table 1:** Clinical data of psoriatic patients.

Characteristics	Psoriasis patients (*N* = 75)	Healthy control (*N* = 52)
Age (years), *M* ± SD	47.1 ± 14.6	48.7 ± 15.2
Sex (male), *n*, %	65, 84.7	43, 82.7
Duration of psoriasis (years), *M* ± SD	20.0 ± 13.6	
Age of psoriasis onset (years), *M* ± SD	27.1 ± 14.6	
Positive family history of psoriasis, *n*, %	28, 37.3	
PASI, *M* ± SD	14.0 ± 9.4	
BSA (%), *M* ± SD	24.0 ± 17.8	
IGA, *n*, %		
Mild	17, 22.7	
Moderate	38, 50.7	
Severe	16, 21.3	
Very severe	4, 5.3	
NAPSI 80, *M* ± SD	18.5 ± 16.5	
DLQI, *M* ± SD	14.0 ± 7.8	

PASI: Psoriasis Area and Severity Index; BSA: Body Surface Area; IGA: Investigator Global Assessment; DLQI: Dermatology Life Quality Index; NAPSI 80: Nail Psoriasis Severity Index 80; M: mean value; SD: standard deviation.

**Table 2:** The distribution of PBMCs and PD-1 expression in the psoriatic patients compared to the control group.

PBMC and PD-1 expression	IU	Psoriasis	Control	Logistic regression analysis (psoriasis versus control)		
		*M* ± SD	*M* ± SD	OR (95% CI)	OR-1 (%)	*p*
CD3^+^	Cells/*μ*L	1146.4 ± 408.5	964.1 ± 339.9	1.001 (1.000, 1.002)	0.1	0.041
	%	59.2 ± 13.0	48.2 ± 17.0	1.051 (1.024, 1.080)	5.1	<0.001
CD4^+^	Cells/*μ*L	687.2 ± 351.4	525.3 ± 253.2	1.001 (1.000, 1.002)	0.1	0.092
	%	59.4 ± 18.2	54.0 ± 17.5	1.017 (0.997, 1.037)	1.7	0.103
CD8^+^	Cells/*μ*L	349.2 ± 215.5	235.6 ± 144.7	1.004 (1.002, 1.006)	0.4	0.001
	%	31.0 ± 16.8	23.3 ± 10.1	1.039 (1.012, 1.068)	3.9	0.005
CD4^+^PD-1^+^	Cells/*μ*L	14.1 ± 11.3	14.7 ± 9.8	0.972 (0.940, 1.004)	−2.8	0.089
	%	2.0 ± 1.2	2.9 ± 1.7	0.631 (0.475, 0.837)	−36.9	0.001
CD8^+^PD-1^+^	Cells/*μ*L	10.2 ± 8.3	17.8 ± 19.7	0.967 (0.939, 0.996)	−3.3	0.024
	%	3.3 ± 2.1	6.6 ± 4.4	0.705, (0.602, 0.824)	−29.5	<0.001

PBMCs: peripheral blood mononuclear cells; PD-1: programmed death 1; *M*: mean value; SD: standard deviation; OR: odds ratio; cells/*μ*L: number of lymphocytes/*μ*L.

**Table 3:** Correlations between clinical data and the distribution of PBMCs and PD-1 expression in the psoriatic patients.

PBMC and PD-1 expression	IU	*r*	Duration of psoriasis (years)	Age of psoriasis onset (years)	PASI	BSA	IGA	NAPSI 80	DLQI
		*p*							
CD3^+^	Cells/*μ*L	*r*	−0.113	−0.043	−0.279	−0.177	−0.181	−0.260	−0.011
		*p*	0.335	0.712	0.015	0.130	0.120	0.024	0.924
	%	*r*	−0.036	−0.030	0.093	0.050	0.057	−0.043	0.136
		*p*	0.758	0.797	0.429	0.670	0.626	0.717	0.243
CD4^+^	Cells/*μ*L	*r*	−0.120	0.085	−0.373	−0.238	−0.297	−0.231	−0.106
		*p*	0.307	0.471	0.001	0.040	0.010	0.046	0.367
	%	*r*	−0.047	0.162	−0.295	−0.205	−0.291	−0.078	−0.159
		*p*	0.687	0.164	0.010	0.078	0.011	0.508	0.174
CD8^+^	Cells/*μ*L	*r*	0.070	−0.190	0.140	0.106	0.164	−0.084	0.147
		*p*	0.548	0.102	0.229	0.366	0.161	0.476	0.207
	%	*r*	0.116	−0.168	0.338	0.253	0.309	0.069	0.154
		*p*	0.320	0.150	0.003	0.029	0.007	0.559	0.186
CD4^+^PD-1^+^	Cells/*μ*L	*r*	−0.117	−0.030	−0.283	−0.129	−0.148	−0.180	−0.018
		*p*	0.317	0.797	0.014	0.269	0.204	0.122	0.882
	%	*r*	−0.034	−0.120	−0.114	−0.001	−0.008	0.017	0.026
		*p*	0.771	0.305	0.330	0.999	0.945	0.884	0.828
CD8^+^PD-1^+^	Cells/*μ*L	*r*	−0.035	−0.257	0.023	−0.036	0.090	−0.103	−0.004
		*p*	0.767	0.026	0.848	0.759	0.445	0.379	0.973
	%	*r*	−0.029	−0.053	−0.022	−0.061	0.047	−0.042	−0.061
		*p*	0.804	0.655	0.852	0.601	0.692	0.721	0.601

PASI: Psoriasis Area and Severity Index; BSA: Body Surface Area; IGA: Investigator Global Assessment; DLQI: Dermatology Life Quality Index; NAPSI 80: Nail Psoriasis Severity Index 80; PBMCs: peripheral blood mononuclear cells; PD-1: programmed death 1; cells/*μ*L: number of lymphocytes/*μ*L.
